# Ginsenoside Compound K Enhances Fracture Healing *via* Promoting Osteogenesis and Angiogenesis

**DOI:** 10.3389/fphar.2022.855393

**Published:** 2022-04-01

**Authors:** Lingli Ding, Song Gu, Bingyu Zhou, Min Wang, Yage Zhang, Siluo Wu, Hong Zou, Guoping Zhao, Zhao Gao, Liangliang Xu

**Affiliations:** ^1^ Key Laboratory of Orthopaedics and Traumatology, Lingnan Medical Research Center, The First Affiliated Hospital of Guangzhou University of Chinese Medicine, Guangzhou University of Chinese Medicine, Guangzhou, China; ^2^ The First Affiliated Hospital, Guizhou University of Traditional Chinese Medicine, Guiyang, China; ^3^ Engineering Laboratory for Nutrition, Shanghai Institute of Nutrition and Health, Chinese Academy of Sciences, Shanghai, China; ^4^ Master Lab for Innovative Application of Nature Products, National Center of Technology Innovation for Synthetic Biology, Tianjin Institute of Industrial Biotechnology, Chinese Academy of Sciences, Tianjin, China; ^5^ CAS Key Laboratory of Quantitative Engineering Biology, Shenzhen Institute of Synthetic Biology, Shenzhen Institute of Advanced Technology, Chinese Academy of Sciences, Shenzhen, China; ^6^ CAS-Key Laboratory of Synthetic Biology, CAS Center for Excellence in Molecular Plant Sciences, Shanghai Institute of Plant Physiology and Ecology, Chinese Academy of Sciences, Shanghai, China; ^7^ Bio-Med Big Data Center, Shanghai Institute of Nutrition and Health, Chinese Academy of Sciences, Shanghai, China; ^8^ State Key Laboratory of Genetic Engineering, Department of Microbiology and Immunology, School of Life Sciences, Fudan University, Shanghai, China; ^9^ Department of Microbiology, The Chinese University of Hong Kong, Hong Kong, Hong Kong SAR, China; ^10^ Er Sha Sports Training Center of Guangdong Province, Guangzhou, China

**Keywords:** CK, fracture healing, Wnt/β-catenin, osteogenesis, angiogenesis

## Abstract

Fractures have an extraordinarily negative impact on an individual’s quality of life and functional status, particularly delayed or non-union fractures. Osteogenesis and angiogenesis are closely related to bone growth and regeneration, and bone modeling and remodeling. Recently Chinese medicine has been extensively studied to promote osteogenic differentiation in MSCs. Studies have found that Ginseng can be used as an alternative for tissue regeneration and engineering. Ginseng is a commonly used herbal medicine in clinical practice, and one of its components, Ginsenoside Compound K (CK), has received much attention. Evidence indicates that CK has health-promoting effects in inflammation, atherosclerosis, diabetics, aging, etc. But relatively little is known about its effect on bone regeneration and the underlying cellular and molecular mechanisms. In this study, CK was found to promote osteogenic differentiation of rat bone marrow mesenchymal stem cells (rBMSCs) by RT-PCR and Alizarin Red S staining *in vitro*. Mechanistically, we found CK could promote osteogenesis through activating Wnt/β-catenin signaling pathway by immunofluorescence staining and luciferase reporter assay. And we also showed that the tube formation capacity of human umbilical vein endothelial cells (HUVECs) was increased by CK. Furthermore, using the rat open femoral fracture model, we found that CK could improve fracture repair as demonstrated by Micro-CT, biomechanical and histology staining analysis. The formation of H type vessel in the fracture callus was also increased by CK. These findings provide a scientific basis for treating fractures with CK, which may expand its application in clinical practice.

## Introduction

Fractures have an extraordinarily negative influence on an individual’s quality of life and functional status. Bone is one of the organs that have the capacity to regenerate. Fracture disrupts bone circulation, leading to necrosis and hypoxia of adjacent bones (Glowacki 1998). Type H vessels, with high expression of Endomucin (Emcn) and CD31, have recently been identified and have the ability to induce bone formation (Peng et al., 2020). Fracture repair usually could restore the damaged bone to its pre-injury cellular composition, structure and biomechanical function, but approximately 10% of fractures do not heal properly (Einhorn and Gerstenfeld 2015). Some alternative therapies can promote fracture healing to prevent delayed healing or non-healing, such as herbal medicine.

Ginseng is a traditional Chinese herb that has been widely used in Asia for thousands of years to keep the physical vigor, improve immunity and resistance to aging, etc. Some ginsenosides have been found to prevent osteoporosis (Liu et al., 2020) and osteoarthritis (Chen et al., 2016), and also improve fracture healing (Gu et al., 2016). Ginsenoside compound K is a metabolite produced by ginsenosides Rb1, Rb2 and Rc through the metabolism of intestinal bacteria *in vivo* (Yang et al., 2015). The metabolic pathway of protopanaxadiol type ginsenosides by human intestinal bacteria is Rb1, Rb2 or Rc→Rd→F2→CK(Zhou et al., 2008), which has also been shown to be same in hydrolytic pathway (Zhou et al., 2018). Rb2 can reduce oxidative damage and bone resorption cytokines, reflecting the ability of anti-osteoporosis (Huang et al., 2014). Treatment of 3T3-L1 cells with CK inhibited adipocyte differentiation and expression of adipocyte-specific genes (Park and Yoon 2012). Meanwhile, a variety of studies have reported that ginsenosides could regulate angiogenesis. For example, ginsenoside-Rg1 has been shown to induce angiogenesis (Kwok et al., 2015). Ginsenoside Rg1 increased the expression of VEGF through PI3K/Akt/mTOR signaling pathway and promoted cerebral angiogenesis after ischemic stroke (Chen et al., 2019a). Ginsenoside F1-induced activation of the IGF-1/IGF1R pathway to promote angiogenesis is an effective approach to alleviate cerebral ischemia (Zhang et al., 2019).

It has been reported that CK could significantly elevate the mRNA expression of genes regulating Wnt/β-catenin signaling, including Wnt10b, Wnt11, Lrp5 and β-catenin (Zhou et al., 2018). Many studies have shown that the Wnt/β-catenin signaling controls bone formation and osteoblast differentiation (Kobayashi et al., 2016; Yuan et al., 2016; Shen et al., 2020). Angiogenesis is an essentially biological process in bone regeneration and is also closely linked to the Wnt/β-catenin signaling pathway (Shi et al., 2020; Shen et al., 2021; Yu et al., 2021). However, it is unknown whether CK is effective on fracture repair, as well as the underlying mechanisms involved. In the present study, we investigated the effects of CK on rat fracture healing, including osteogenic differentiation and angiogenesis, and elucidated its potential regulation of Wnt/β-catenin signaling pathway.

## Materials and Methods

### Reagents and Antibodies

CK was provided by Zhejiang Hongguan Bio-pharma Co., Ltd., and dissolved in DMSO, and diluted in PBS. Modified Eagle’s Medium of Alpha (α-MEM), Dulbecco’s Modified Eagle Medium/Nutrient Mixture F-12 (DMEM/F-12), fetal bovine serum (FBS), and penicillin/streptomycin were purchased from Gibco (United States). Beta-glycerolphosphate, dexamethasone, ascorbic acid phosphate, Safranine O, and Fast Green were purchased from Sigma (United States). Alizarin Red S was purchased from Solarbio (Beijing, China). Cell Counting Kit-8 (CCK-8) was purchased from Beyotime (Beijing, China). NucleoZOL reagent, Reverse Transcription Kit and SYBR-Green Master Mix were supplied by Takara (Japan). Hematoxylin-eosin (H&E) was purchased from biosharp (China). Primary antibodies against CD31, β-catenin, and DAPI were supplied by Santa Cruz Biotechnology (United States); Primary antibodies anti-CTSK, anti-ALP, anti-OPG, anti-RANKL, anti-Runx2, and anti-OPN were purchased from Bioss (China); Anti-GAPDH, and DAPI were obtained from Abcam (United States). Secondary antibodies HRP-conjugated Goat Anti-Rabbit IgG, Goat anti-Mouse IgG (H + L), Rabbit Anti-Rabbit IgM/Cy3 and Rabbit Anti-Mouse IgM/FITC were obtained from Bioss (China). Dual-Luciferase Reporter Assay System was supplied by Promega Company (United States). Matrigel was purchased from Becton Dickinson (United States).

### RNA Extraction and qRT-PCR

After inducing differentiation for 3 days in 12-well plates with osteogenic induction medium, the total RNA was extracted using NucleoZOL, and cDNA was obtained from total RNA using a Reverse Transcription Kit. Next, qRT-PCR was performed using SYBR Green qPCR Master Mix. The relative gene expression was calculated by the 2^–ΔCT^ method, and GAPDH was used as a reference for normalization. The primers were purchased from Invitrogen (United States) and primer sequences are shown in Table 1.

**TABLE 1:** Primers used for RT-PCR.

Traget gene	Sequence (5′-3′)
GAPDH	F,AGGTCGGTGTGAACGGATTTG
	R,TGTAGACCATGTAGTTGAGGTCA
OPN	F,AGCAAGAAACTCTTCCAAGCAA
	R,GTGAGATTCGTCAGATTCATCCG
OCN	F,GGTGGCTTCCGAAGGATTGTC
	R,CCCCCTGATGGGTTGTCAC
OSX	F,ATGGCGTCCTCTCTGCTTG
	R,TGAAAGGTCAGCGTATGGCTT
ALP	F,GCAAGGGTGAGGAGGGGTA
	R,CCTCTGAAGGCATTTCATAAGCC

### Bone Marrow Mesenchymal Stem Cells Isolation and Culture

The method of rat bone marrow mesenchymal stem cells (BMSCs) isolation and cultivation has been described previously (Shen et al., 2018). BMSCs were isolated from Sprague-Dawley rats (male, 2 weeks old, 30–40 g) in a sterile environment. Rats were euthanized and the bone marrow of the bilateral femoral was flushed out with serum-free α-MEM to obtain a single-cell suspension. Flushed bone marrow cells was centrifuged at 1200 rpm for 6 min, the supernatant liquid was removed, and the cell pellets were resuspended in α-MEM supplemented with 10% fetal bovine serum, 1% penicillin and streptomycin. The medium was discarded after 72 h of primary culture and then changed once every 3 days. Upon 80—90% confluence, the adherent cells were further expanded with trypsin. The medium was changed several times to obtain pure BMSCs. Cells from passages 3 to 5 were used in the study.

### Cell Counting Kit-8 Assay

The cell viability of CK on BMSCs was assessed using a Cell Counting Kit-8 (CCK-8) kit (C0037, Beyotime Biotechnology Co., Ltd., Shanghai, China). BMSCs or HUVECs were seeded in a 96-well plate with 5000 cells in each well and cultured without or with CK (0, 2.5,5, 10, 20, 30 and 40 μM) for 48 and 72 h. Subsequently, CCK-8 reagent was added to each well and the plates were incubated for 1 h at 37°C. The Optical density (OD) of the samples was measured at 450 nm with a spectrophotometric microplate reader (Xianke Instruments, Shanghai, China). The experiment was independently repeated 3 times.

### Animal Experiments

8 week-old male Sprague Dawley (SD) rats (220 ± 10 g, *n* = 18) were purchased from the Guangzhou Medicine Laboratory Animal Center. The animals were housed in the First Affiliated Hospital of Guangzhou University of Chinese medicine animal center. All experimental methods were approved by the Animal Care and Use Committee of Guangzhou University of Traditional Chinese Medicine. All rats were fed with standard chow and free access to water with a 12 h light-dark cycle (24 ± 1°C). An open femoral fracture model with internal fixation was established. In brief, the procedure was performed under general anesthesia (pentobarbital sodium 100 mg/kg, intraperitoneally) and aseptic conditions. The right femur was exposed, and transverse osteotomy was performed with a hand saw to create a gap size of 2 mm. A K-wire (diameter: 1.2 mm, Stryker Ltd., United States) was inserted into the right femoral bone marrow cavity to fix the fracture. The rats were randomly assigned to the following two groups: fracture + PBS (*n* = 9, 100 μl/day), fracture + CK (*n* = 9, CK = 500 μM, 100 μl every other day). 5 days after the surgery, CK or PBS was locally injected at the fracture sites every other day for 4 weeks. Then the animals were sacrificed and the right femurs were collected for further analysis.

### Histology

The femurs were harvested, fixed in 10% neutral formalin for 24 h, decalcified in 10% Ethylene Diamine Tetraacetic Acid (EDTA) for 21 days, dehydrated and then embedded in paraffin. After cutting into 5-μm-thick sections and dewaxing in xylene and rehydration in a decreasing alcohol gradient and distilled water, samples were processed for hematoxylin-eosin (H&E), and Safranine O-Fast Green (SO-FG) staining. For immunohistochemical staining, the sections were incubated in 0.3% hydrogen peroxide for 20 min and antigen retrieval in 0.01 M citrate buffer at 60°C for 30 min, and blocking with 5% g BSA in PBS for 1 h and then incubated overnight at 4°C with a primary antibody. The sections were then incubated with the secondary antibodies for 1 h at 37°C, counterstained with hematoxylin, and visualized using an HRP-streptavidin system. The primary antibodies used in this study included anti-OPG, anti-RANKL, anti-OPN and anti-ALP antibodies. For immunofluorescence staining, the slides were incubated in antigen retrieval in 0.01 M citrate buffer, and blocking with 5% g BSA in PBS for 1 h and then incubated overnight at 4°C with a primary antibody. The sections were then incubated with the fluorescent secondary antibodies for 1 h at 37°C. The primary antibodies used in this study included anti-Runx2, anti-Endomucin, anti-CD31 and anti-β-catenin antibodies. DAPI staining was carried out to stain the nuclei. Images were acquired with the fluorescence microscope (Olympus, IX73 L, United States).

### Microcomputer Tomography

Microcomputer tomography (micro-CT) examination was applied for the fractured femurs. Samples were scanned by Skyscan 1176 micro-CT scanner (Bruker micro-CT, Kontich, Belgium), with a source voltage of 80 kV, current of 114 μA, Al 0.5 mm filter and 10.5 μm isotropic resolution. The fractured callus sites were defined as the volume of interest. The bone volume/tissue volume (BV/TV), mean volumetric bone mineral density (BMD), and Callus Volume were measured. Three dimensional images were generated using CTvol software (Bruker micro-CT, Kontich, Belgium).

### Tube Formation Assay

Tube formation assay was performed as previously reported (Lin et al., 2019). The wells of the 12-well plate were coated with Matrigel and incubated for 30 min hBMSCs (5000 cells/well) were treated with or without CK (10 μM), and co-cultured with HUVECs (10^5^ cells/well). DMEM/F12 basal medium containing 2% FBS (Thermo Fisher Scientific) was used. Plates were incubated at 37°C, 5% CO_2_ for 8 h. Then the tube formation was observed using a microscope. Tubes were then assessed through an inverted fluorescent microscope at 10×(Olympus). Image J with the Angiogenesis Analyzer plugin were used to quantify the tube length and branch points of tube networks. Images taken at 5× magnification.

### Luciferase Reporter Assays

Experiments were performed as described previously (Ren et al., 2019). Briefly, 293FT cells were seeded on 24-well plates and allowed to grow to 80% confluence. Cells were then transfected with TOPflash (500 ng) and Renilla reporter plasmid pRL-CMV (100 ng) using Lipofectamine 8000. 24 h after transfection, cells were treated with CK (10 μM) for 24 h. The luciferase activity was measured using a GloMax™ 20/20 single-tube luminometer (Promega, Madison, WI, United States).

### Three-Point Bending Biomechanical Testing

Three-point bending biomechanical testing was performed as previously reported (Lin et al., 2019). Fractured femurs were tested to failure with a constant displacement rate of 4 mm/min by a 3-point bending device (H25KS; Tinius Olsen, United Kingdom). The fractured femur was loaded in the front and back directions, and the span of the two support points was set as 10 mm. The force loading point was set at the fracture site. After testing, ultimate load to failure and energy absorbed to failure were recorded and analyzed by the QMAT software.

### Statistical Analysis

We applied GraphPad Prism 5 for comparison. Quantitative data were expressed as mean ± standard deviation (SD). Statistics were analyzed by t-test for two-group comparison, and One-way or two-way analysis of variance (ANOVA) for multi-comparison between groups. We used Tukey’s post hoc multiple comparisons test as the posttest method for ANOVA. P values <0.05 were considered statistically significant.

## Results

### CK Enhanced Osteogenic Differentiation and Angiogenesis *In Vitro*

**FIGURE 1:**
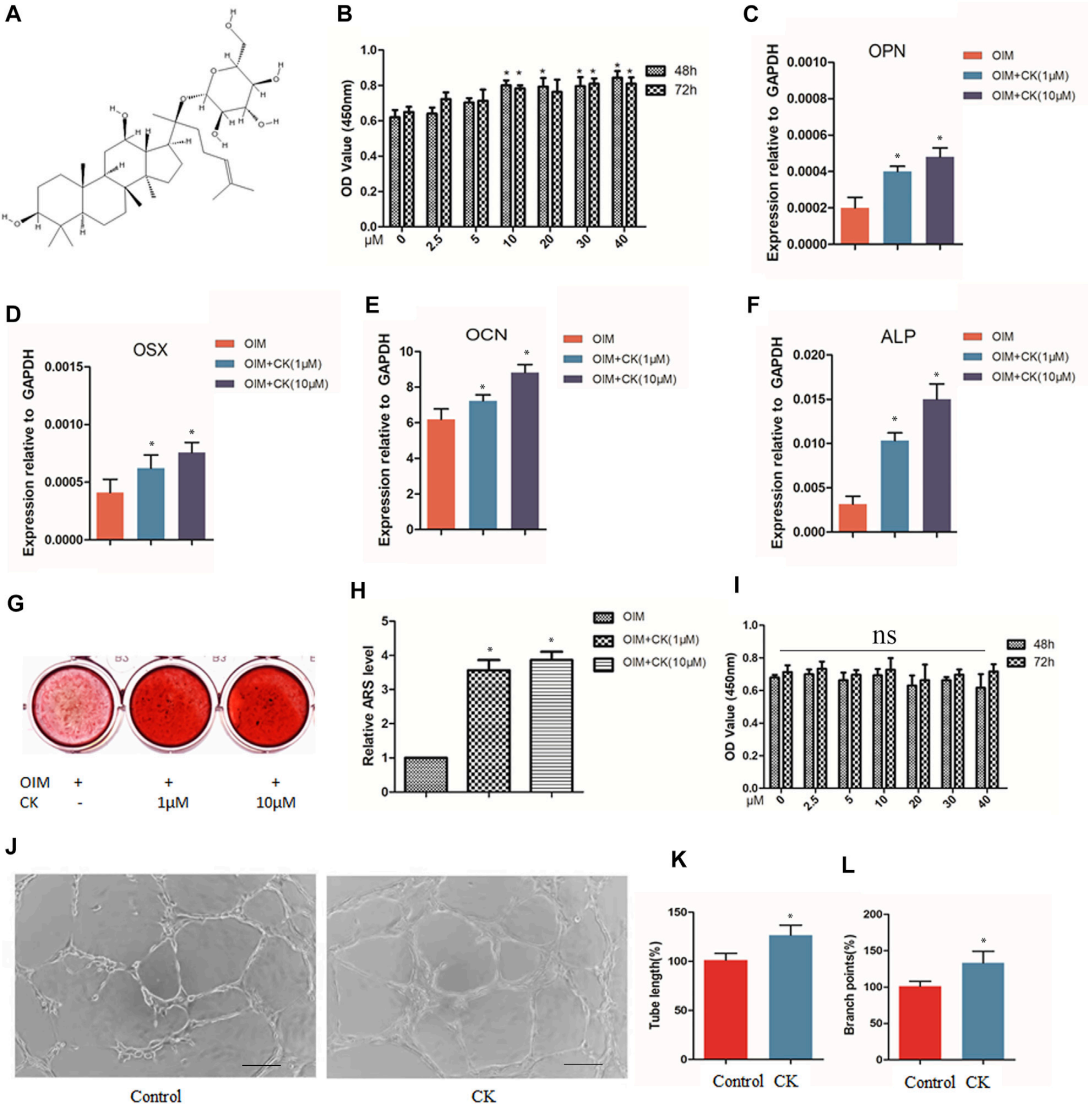
CK enhanced cell viability, osteogenesis and angiogenesis *in vitro*. **(A)** The chemical structure depiction of CK. **(B)** Optical density (OD) values indicating the effects of CK treatment for 48 h and 72h on cell viability of BMSCs, *p < 0.05, *n* = 3, compared with 0 μM CK group. **(C–F)** Real-time PCR results of osteogenesis-related genes at 3 days treated with different concentrations of CK in OIM, *p < 0.05, *n* = 3, compared with OIM group. **(G)** Representative staining images and **(H)** Quantification of Alizarin Red S of BMSCs incubated with different concentrations of CK in OIM for 14 days, *p < 0.05, *n* = 3, compared with OIM group. **(I)** Optical density (OD) values indicating the effects of CK treatment for 48 h and 72h on cell viability of HUVECs, *p < 0.05, *n* = 3, compared with 0 μM CK group. **(J)** Representative images of tube formation assay and **(K-L)** Quantitative analysis of tube length and branch points in Matrigel MSCs and HUVEC co-culture system, Scale bars < 100 μm, *p < 0.05, compared with the Control group, *n* = 3.

The chemical structure depiction of CK was shown in Figure 1A. BMSCs were cultured without or with CK (0, 2.5,5, 10, 20, 30 and 40 μM) for 48 and 72 h. The CCK-8 assay results showed that CK exhibited no obvious cytotoxicity to BMSCs even at the high concentration of 40µM, and CK at 10–40 µM increased the cell viability of BMSCs (Figure 1B). To investigate the effect of CK on osteogenic differentiation of BMSCs, BMSCs were cultured in the osteogenic induction medium (OIM) with or without CK at various concentrations (0, 1, and 10 μM) for 3 and 14 days. At the third day of osteogenic induction, the expression of osteogenesis-related genes was detected and it was found that the RNA levels of osteopontin (OPN), alkaline phosphatase (ALP), osteocalcin (OCN) and osterix (OSX) were significantly up-regulated by CK (Figures 1C–F). Furthermore, Alizarin Red S staining showed that CK significantly increased the formation of calcium deposits after 14 days of induction (Figures 1G,H). As 10 μM CK showed the best effect, it was used for the following *in vitro* experiments.

HUVECs were cultured without or with CK (0, 2.5,5, 10, 20, 30 and 40 μM) for 48 and 72 h. The CCK-8 assay showed that CK exhibited no obvious cytotoxicity to HUVECs (Figure 1I). Some studies have reported MSCs can stimulate migration and angiogenesis of HUVECs (Chiang et al., 2018; Liu et al., 2019). To investigate the effect of CK on angiogenesis in the presence of MSCs, the HUVEC cells were co-cultured with BMSCs treated with or without CK (10 µM), and the tube length and branch points were evaluated (Figures 1J–L). We could observe that the tube formation capacity of HUVEC was increased by CK. The result demonstrated that CK enhanced angiogenesis *in vitro* compared with the control group.

### CK Activated Wnt/β-Catenin Signaling Pathway in Bone Marrow Mesenchymal Stem Cells

**FIGURE 2:**
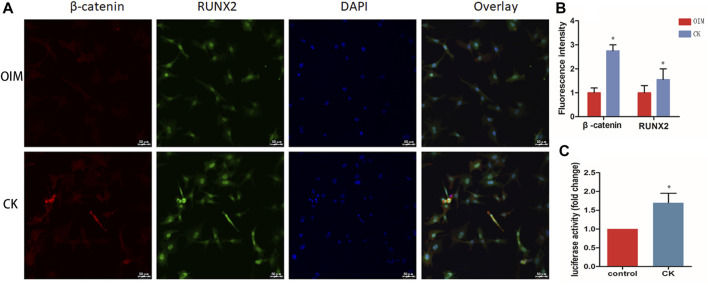
CK increased β-catenin and Runx2 expression in BMSCs. **(A)** Representative images and **(B)** Quantification of immunofluorescence staining of β-catenin and Runx2 treated with 10 μM CK for 24 h. Scale bars = 50 μm, *p < 0.05, compared with the OIM group, *n* = 3 **(C)** The TOPflash luciferase activity was measured in BMSCs after treatment of CK (10 μM). *p < 0.05, compared with the Control group, *n* = 3.

It is well known that the Wnt/β-catenin signaling has been shown as an important regulatory pathway in the osteogenic differentiation of mesenchymal stem cells (Kim et al., 2013). To verify whether the Wnt/β-catenin signaling pathway is activated upon CK treatment in BMSCs, we performed immunofluorescence staining to detect the level of β-catenin and its co-location with Runx2 in rBMSCs. Immunofluorescence analysis revealed more nuclear translocation of β-catenin and an increased expression of Runx2 in the CK (10 μM) group, compared with the OIM group (Figures 2A,B). Additionally, the TOP flash assay was used to evaluate the effect of CK (10 μM) on the activation of the Wnt/β-catenin signaling pathway. After 24 h of stimulation, the luciferase activity was significantly increased by CK (Figure 2C).

### CK Improved Fracture Healing

**FIGURE 3:**
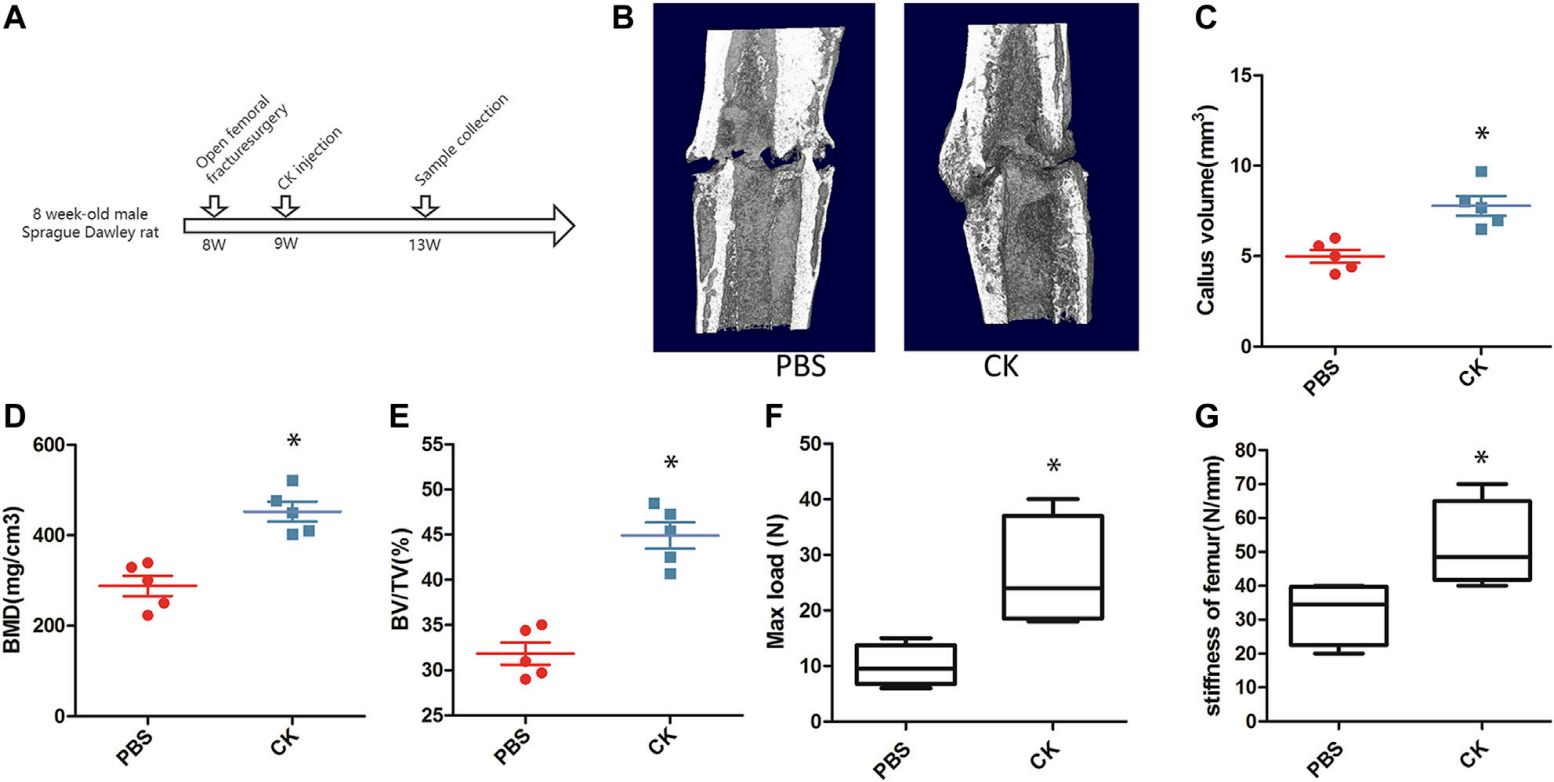
CK accelerated the progression of fracture healing. **(A)** Schematic illustration of time points of animal modeling and sample collection. **(B)** Representative 3-dimensional micro-CT images of femurs in each group. **(C–E)** Quantitative analysis of parameters, including CV, BMD, and BV/TV, *p < 0.05, compared with the PBS group, *n* = 3 **(F–G)** Biomechanical properties of the fractured bones by 3-point bending test, *p < 0.05, compared with the PBS group, *n* = 6.

We further conducted an open femoral fracture model to evaluate whether CK could accelerate fracture healing in rats. The time points of animal modeling and sample collection were shown in Figure 3A. The 3-dimensional images of the femurs obtained by micro-CT analysis showed that the fracture gap was almost filled by new bone in the CK-treated rats at 4w post-fracture, compared with that of the PBS group (Figure 3B). The BMD, BV/TV and callus volume at femoral callus sites of CK-treated rats were significantly higher than those in the PBS group (Figures 3C–E). In addition, results of biomechanical testing confirmed a much stronger biomechanical property in the femoral bones of CK- treated group than those of the PBS group (Figures 3F,G).

**FIGURE 4:**
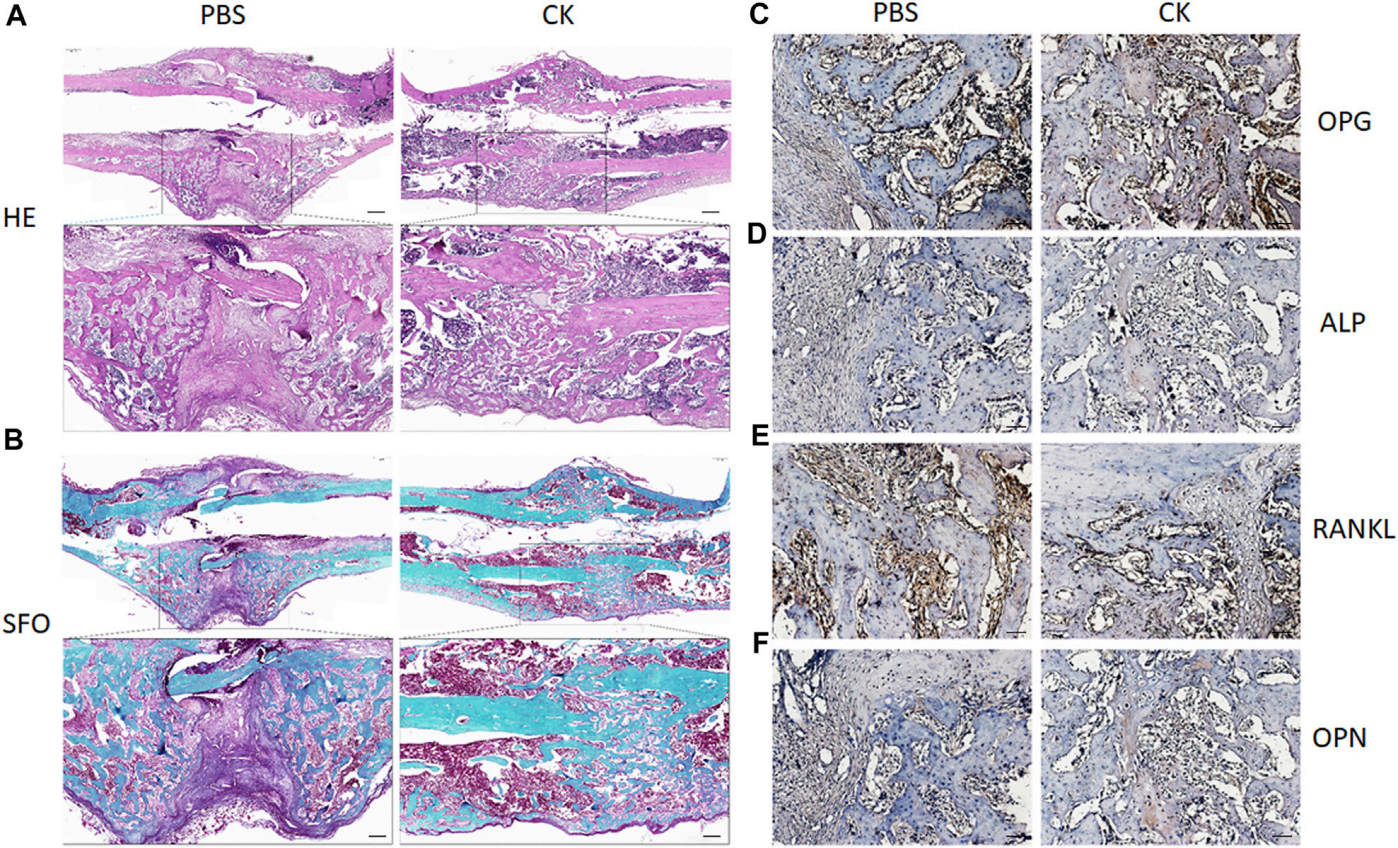
H&E, Safranin O-Fast Green, and immunohistochemical examination of fracture callus. **(A)** Representative images of H&E staining of fracture calluses in PBS and CK groups. Insets indicate the regions shown in the enlarged images (lower). Scale bar: 500 μm. **(B)** Representative images of Safranin O-Fast Green staining of fracture calluses in PBS and CK groups. Insets indicate the regions shown in the enlarged images (lower). **(C–F)** Representative images of immunohistochemical analysis of OPG, ALP, RANKL and OPN of fracture calluses in PBS and CK groups. Scale bar = 50 μm.

Furthermore, H&E and SO-FG staining of the callus showed varying amounts of newly formed trabecular bone, cartilage tissue and fibrous-like tissue. Callus of CK-treated rats exhibited enhanced bone regeneration after 4 weeks, in comparison with the PBS group, which was evidenced by more neo-formed trabecular bone and less cartilaginous and fibrous-like tissue in the CK group (Figures 4A,B). Additionally, higher expression of OPG, OCN, ALP and lower expression of RANKL within the callus areas of the CK group was confirmed using immunohistochemical analysis (Figures 4C–F).

### CK Promoted H Type Vessel Formation *In Vivo*

**FIGURE 5:**
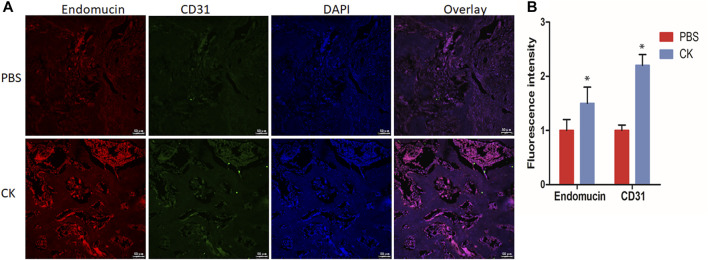
CK regulated H-type vessel formation in fracture callus. **(A)** Representative immunofluorescence double staining and **(B)** Quantification of CD31 (green), EMCN (red) of fracture calluses in PBS and CK groups, Scale bars = 50 μm, *p < 0.05, compared with the PBS group, *n* = 3.

In addition, the H-type vessel was observed *in vivo* in sections of femur. The CD31 and Emcn double immunofluorescent staining revealed a greater population of CD31^hi^Emcn^hi^ cells within the callus of the CK group, compared with the PBS group (Figures 5A,B).

### CK Up-Regulated the β-Catenin Expression in the Fracture Callus

**FIGURE 6:**
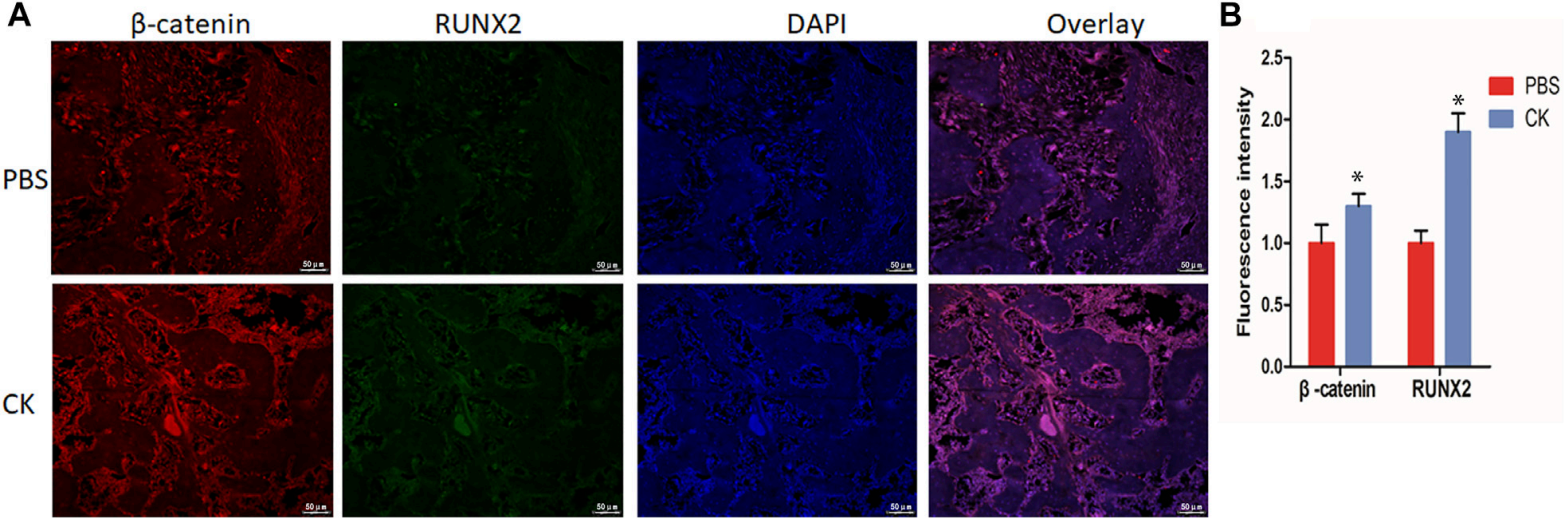
CK up-regulated β-catenin and Runx2 in fracture callus. **(A)** Immunofluorescence staining images of β-catenin and Runx2 in PBS and CK groups and **(B)** Quantification of the immunofluorescence density of β-catenin and Runx2 of fracture calluses in PBS and CK groups, Scale bars = 50 μm, *p < 0.05, compared with the PBS group, *n* = 3.

To detect the expression of β-catenin in fracture callus treated with CK, we performed immunofluorescent staining to detect the level of β-catenin and Runx2 in fracture callus. Immunofluorescence analysis revealed a higher expression of β-catenin and Runx2 within the callus of the CK group, compared with PBS group (Figures 6A,B), which is consistent with the *in vitro* experiment.

## Discussion

Osteogenic differentiation of MSCs is the principal mechanism of bone regeneration and fracture repair. It has been reported that the expressions of alkaline phosphatase (ALP), type I collagen (COL-I) and mineralization were significantly increased after CK treatment in H_2_O_2_-stimulated MC3T3-E1 cells (Kang et al., 2016). Studies have shown that CK improved the biocompatibility and morphology of microsphere scaffolds without affecting the biocompatibility, and CK impregnated porous microsphere scaffold system may be applicable as a promising microsphere scaffold for bone regeneration (Thangavelu et al., 2020). In this study, we found that CK promoted the osteogenic differentiation of BMSCs. Our results demonstrated that CK enhanced the mineralization and mRNA expression of osteogenic markers in rat BMSCs, including ALP, Runx2, OPN and OCN. Fracture healing is closely related to the number and activity of BMSCs near the fracture site (Gu et al., 2016). What’s more, human umbilical vein endothelial cells (HUVECs) play an important role as a model system for studying the regulation of endothelial cell function and angiogenesis. It is well known that both osteogenesis and angiogenesis are integrated parts of bone regeneration (Huang et al., 2015). Interestingly, our results showed CK increased tube formation when HUVEC and BMSCs were co-cultured. Therefore, the *in vitro* experiments indicated that CK might up-regulate osteogenesis coupled with angiogenesis in bone regeneration.

CK is an initial bacterial metabolite of ginsenoside Rb1, which has many advantages of pharmacological properties, such as anti-cancer, anti-inflammatory, anti-aging, anti-allergenic, anti-diabetic, and anti-diabetic (Yoon et al., 2007; Yang et al., 2015; Wang et al., 2017; Chen et al., 2019b; Yin et al., 2021). In open femoral fracture rats, micro-CT examination showed that callus growth in rats treated with CK was substantially faster than that in control rats after fracture, and BMD, BV/TV, and callus volume were significantly increased in the CK-treated group. In addition, biomechanical testing confirmed a much stronger biomechanical property in the femoral bones of CK-treated rats than those of PBS-treated rats. The results of H&E, Safranin-O/Fast Green and IHC staining revealed that, compared with the PBS group, fracture callus in the CK treatment group had a significantly higher proportion of trabecular bone and better fracture healing but a much lower proportion of fibers and cartilage components inside the callus.

Recent studies have revealed that ginsenoside CK regulates multiple signaling pathways, such as PI3K/mTOR/p70S6K1, HIF-1α/NF-κB, Nrf2/Keap1, RhoA/ROCKs/YAP, and PI3K-Akt signaling pathway (Li et al., 2018; Yang et al., 2019; Chen et al., 2020; Tian et al., 2021; Zhang et al., 2021). Wnt/β-catenin signaling pathway is important not only in the growth and development of mineralized tissues, but also in regulating the skeletal response to load and unloading and the vitality and health of adult and aging bones (Duan and Bonewald 2016). Wnt/β-catenin signaling pathway has been widely reported in the regulation of osteogenesis and angiogenesis (Jiang et al., 2015; Shen et al., 2020). Runx2 is a master transcription factor governing osteogenesis (Vimalraj et al., 2015). In the present study, we found that the expression of Runx2 and β-catenin was significantly elevated *in vivo* by immunofluorescent staining, which is consistent with the *in vitro* result. Additionally, the luciferase activity further verified that CK activated β-catenin expression.

The vascular system is a major source of oxygen, nutrients, hormones, neurotransmitters and growth factors to bone cells and is essential for bone development, regeneration and remodeling (Filipowska et al., 2017). Bone regeneration is closely related to angiogenesis and impaired angiogenesis often leads to failure of fracture healing. H-type vessels, highly positive for CD31 and Endomucin, could mediate local growth of the vascular system, combine angiogenesis with osteogenesis by mediating the selective location of Osterix positive cells around blood vessels and the differentiation of these bone progenitor cells (Kusumbe et al., 2014; Ramasamy et al., 2014). In this study, we proved that CK could stimulate type H vessel formation, and promote bone formation in fracture rats.

## Conclusion

Taken together, our study suggested that Wnt/β-catenin signaling contributed to the enhancement in the coupling of osteogenesis and angiogenesis induced by CK treatment during fracture healing. CK may serve as an effective component of Ginseng in bone tissue regeneration. However, many more potential mechanisms remain undiscovered. Further experiments or clinical trials are needed to be conducted to expand its clinical application.

## Data Availability

The original contributions presented in the study are included in the article/Supplementary Material, further inquiries can be directed to the corresponding authors.
