# Corrigendum: Ginsenoside Compound K Enhances Fracture Healing *via* Promoting Osteogenesis and Angiogenesis

**DOI:** 10.3389/fphar.2022.952598

**Published:** 2022-06-16

**Authors:** Lingli Ding, Song Gu, Bingyu Zhou, Min Wang, Yage Zhang, Siluo Wu, Hong Zou, Guoping Zhao, Zhao Gao, Liangliang Xu

**Affiliations:** ^1^ Key Laboratory of Orthopaedics and Traumatology, Lingnan Medical Research Center, The First Affiliated Hospital of Guangzhou University of Chinese Medicine, Guangzhou University of Chinese Medicine, Guangzhou, China; ^2^ The First Affiliated Hospital, Guizhou University of Traditional Chinese Medicine, Guiyang, China; ^3^ Engineering Laboratory for Nutrition, Shanghai Institute of Nutrition and Health, Chinese Academy of Sciences, Shanghai, China; ^4^ Master Lab for Innovative Application of Nature Products, National Center of Technology Innovation for Synthetic Biology, Tianjin Institute of Industrial Biotechnology, Chinese Academy of Sciences, Tianjin, China; ^5^ CAS Key Laboratory of Quantitative Engineering Biology, Shenzhen Institute of Synthetic Biology, Shenzhen Institute of Advanced Technology, Chinese Academy of Sciences, Shenzhen, China; ^6^ CAS-Key Laboratory of Synthetic Biology, CAS Center for Excellence in Molecular Plant Sciences, Shanghai Institute of Plant Physiology and Ecology, Chinese Academy of Sciences, Shanghai, China; ^7^ Bio-Med Big Data Center, Shanghai Institute of Nutrition and Health, Chinese Academy of Sciences, Shanghai, China; ^8^ State Key Laboratory of Genetic Engineering, Department of Microbiology and Immunology, School of Life Sciences, Fudan University, Shanghai, China; ^9^ Department of Microbiology, The Chinese University of Hong Kong, Hong Kong, Hong Kong SAR, China; ^10^ Er Sha Sports Training Center of Guangdong Province, Guangzhou, China

**Keywords:** CK, fracture healing, Wnt/β-catenin, osteogenesis, angiogenesis

In the original article, there was a mistake in Figure 3A as published. Figure 3A is a schematic diagram of the time points of animal modeling and samples, in which the model and time points were incorrectly marked. The corrected Figure 3 appears below.

**FIGURE 3:**
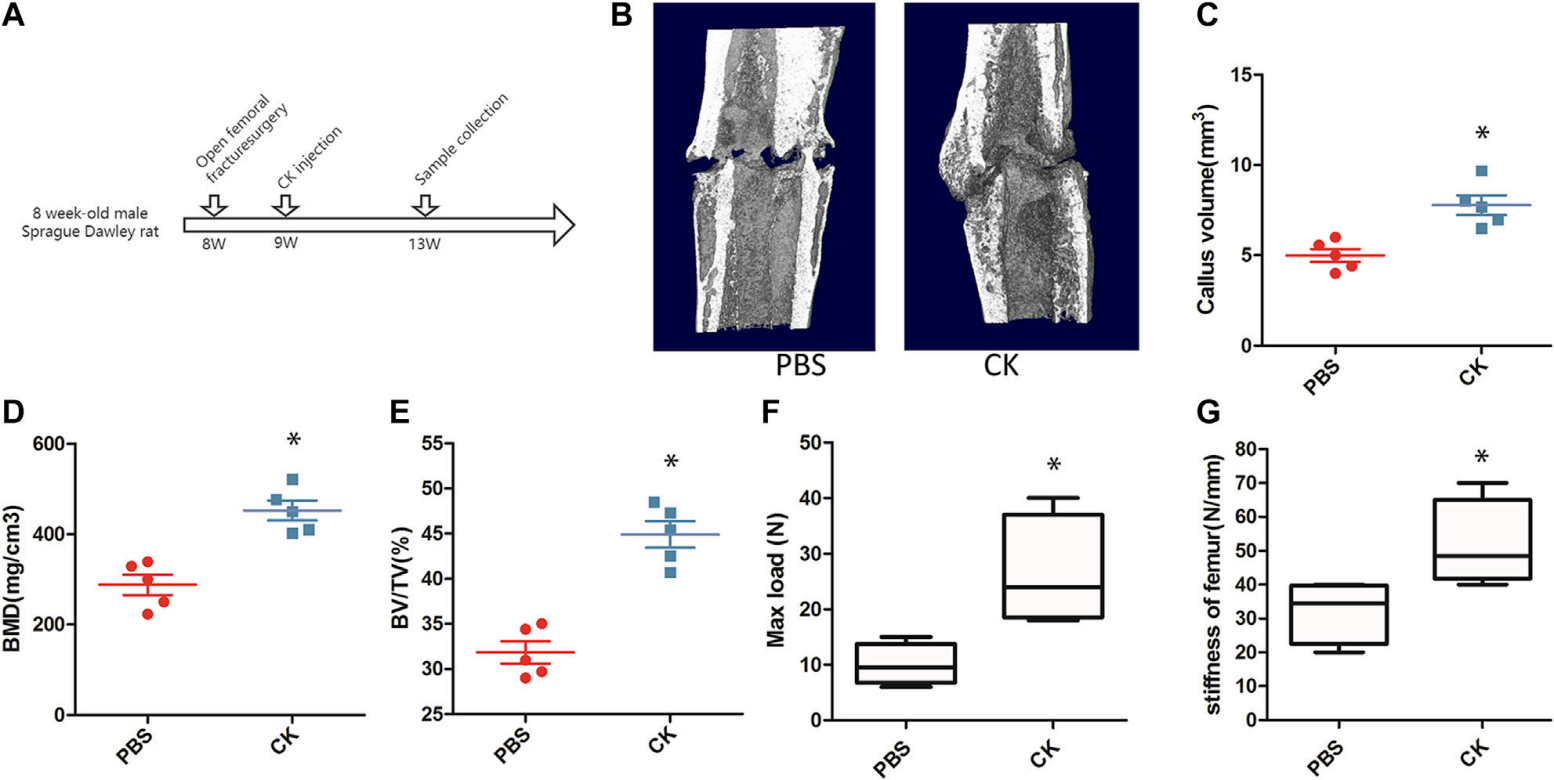
CK accelerated the progression of fracture healing. **(A)** Schematic illustration of time points of animal modeling and sample collection. **(B)** Representative 3-dimensional micro-CT images of femurs in each group. **(C–E)** Quantitative analysis of parameters, including CV, BMD, and BV/TV, *p < 0.05, compared with the PBS group, *n* = 3 **(F–G)** Biomechanical properties of the fractured bones by 3-point bending test, *p < 0.05, compared with the PBS group, *n* = 6.

The authors apologize for this error and state that this does not change the scientific conclusions of the article in any way. The original article has been updated.

